# Supplementary material for: Rapid spatial cognition in mice, with and without neocortex and hippocampus
Source: bioRxiv. 2026 Sep 29:2026.08.30.747945. Preprint. [Version 2] doi: 10.64898/2026.08.30.747945 (PMC13648374; doi:10.64898/2026.08.30.747945)
Supplement: Supplementary file 1 [file NIHPP2026.08.30.747945v2-supplement-7.pdf]

bioRxiv preprint doi: https://doi.org/10.64898/2026.08.30.747945; this version posted September 29, 2026. The copyright holder for this preprint (which was not certified by peer review) is the author/funder, who has granted bioRxiv a license to display the preprint in perpetuity. It is made available under a CC-BY 4.0 International license.

6 Supplementary Materials

6.1 Supplementary figures

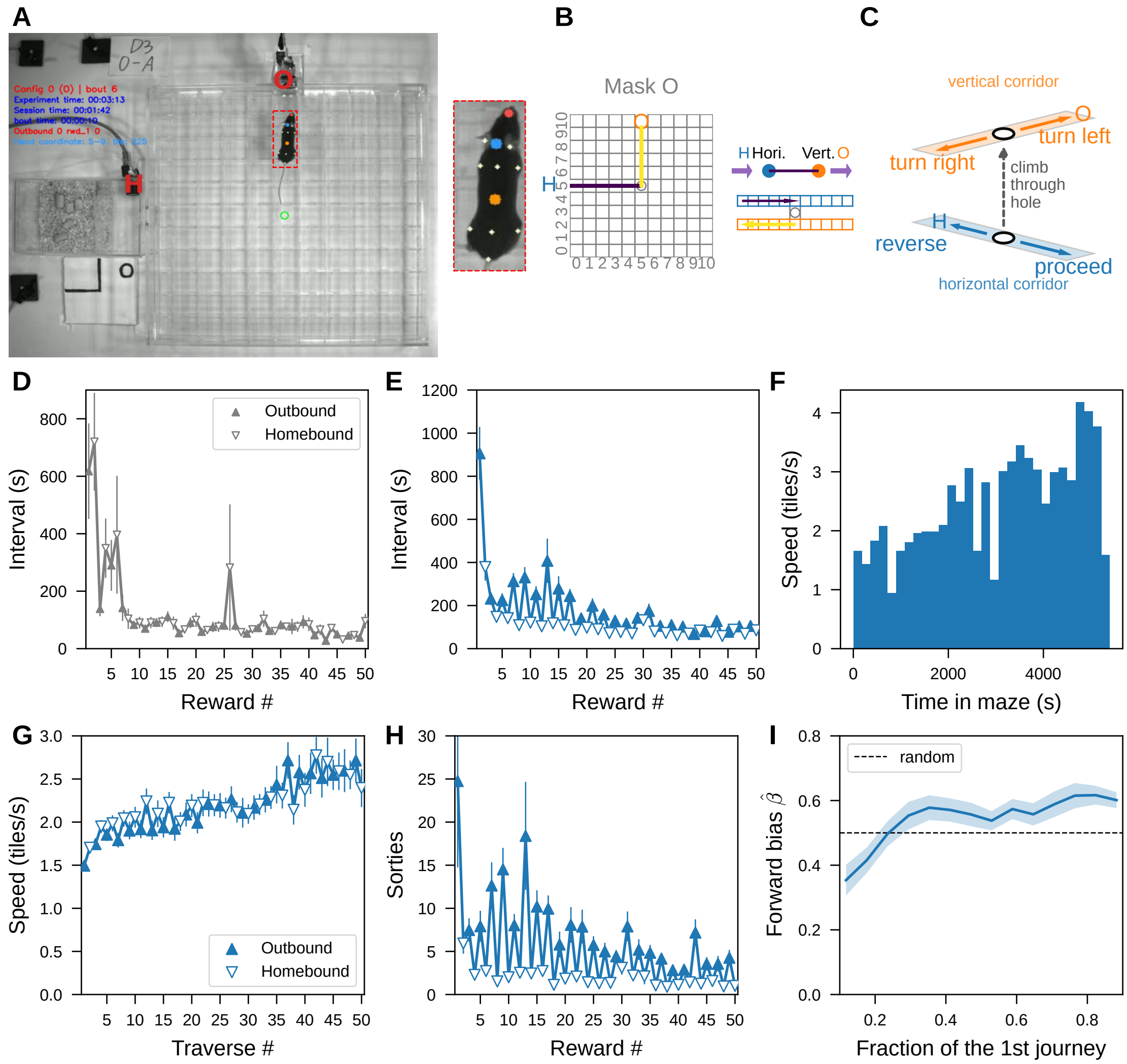

Supplementary Figure S1: **Supplementary results for Day 1 experiments. A-D. Learning Mask O. A.** Top view of the arena with Mask O. A mouse cage is attached to the left entrance; it includes the Home water port (H). The Out water port (O) is connected to the top entrance. The mouse was tracked with 12 keypoints (right), with the head center (blue) used for trajectory segmentation (Section 5.6.2). **B.** Left: Top view of the shortest path in the Mask O maze. The tile coordinates are indexed from zero at the bottom left corner. Right: Graph representation of Mask O. Top: the graph of Mask O in corridors. Bottom: the hole segmented the corridors into a left turn from H to O. **C.** Four decisions can be made at a hole: from the horizontal (blue) corridor, the mouse can climb up the hole and make a left or right turn in the vertical corridor (orange arrows), or stay in the corridor to proceed or reverse from the hole (blue arrows). **D.** Reward intervals (in session time) on Mask O (25 mice) shortened quickly after the first two rewards. **E-I. Learning Mask A.** Lines and errorbars (or shade) show mean ± SE across animals. **E.** Reward intervals in the Mask A maze (25 mice). **F.** Speed in the Mask A maze by Wildtype 1 in Fig. 1D (bin size = 3 minutes and one tile is 1.5 inches). **G.** Traverse speed by all mice in Fig. 1G (25 mice). **H.** Number of sorties between rewards. **I.** Forward bias ($\hat{\beta}$, Section 5.7.1) of the mice before the first reward. Line smoothed with a moving average over 0.2 of the journey.

bioRxiv preprint doi: https://doi.org/10.64898/2026.08.30.747945; this version posted September 29, 2026. The copyright holder for this preprint (which was not certified by peer review) is the author/funder, who has granted bioRxiv a license to display the preprint in perpetuity. It is made available under a CC-BY 4.0 International license.

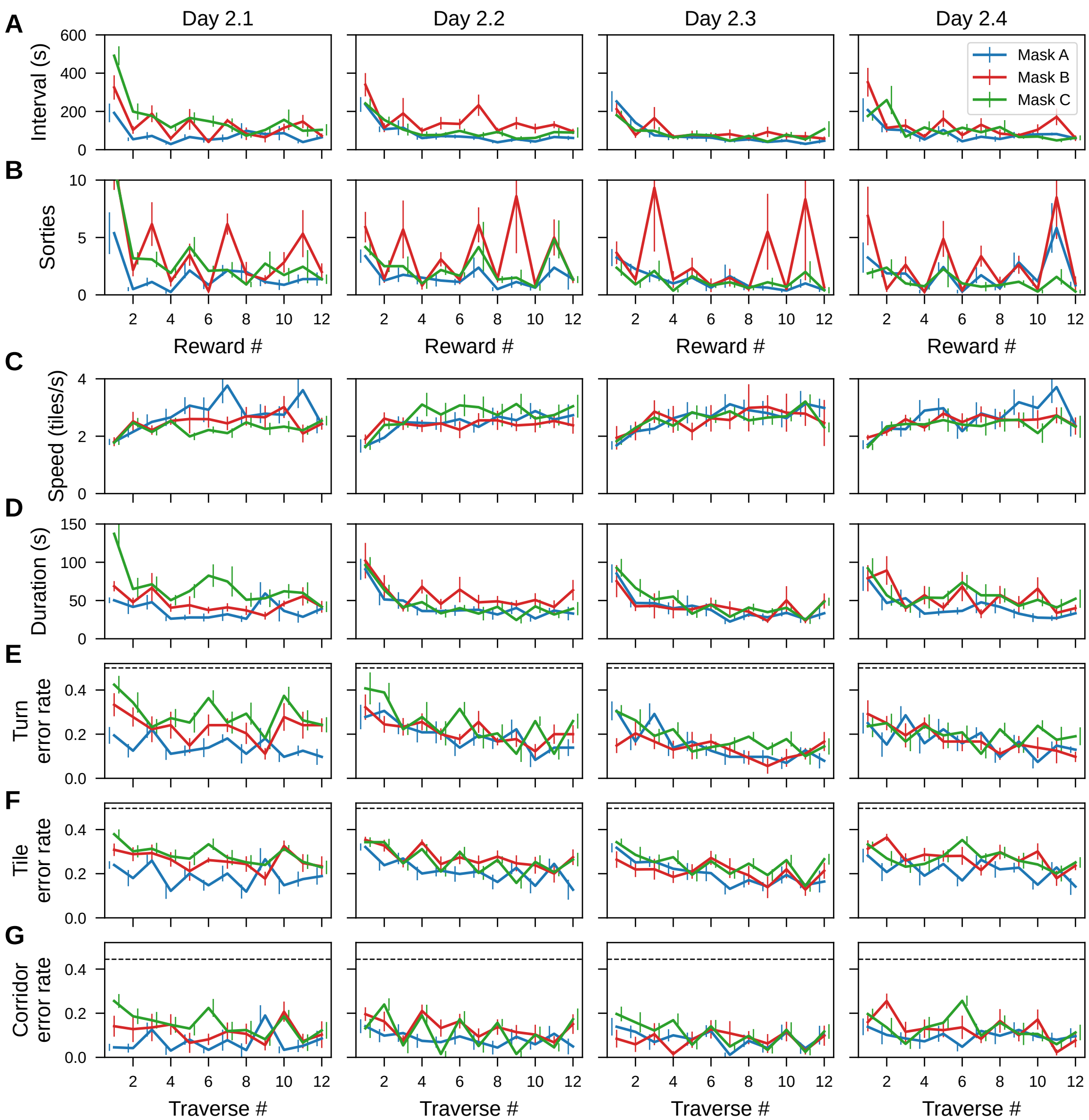

Supplementary Figure S2: **Additional metrics of performance on Day 2.** Lines and errorbars show mean ± SE across animals. Dashed lines, chance error rates. **A.** Reward intervals across all four sessions, grouped by masks. **B.** Number of sorties between rewards. **C.** Traverse speed (tiles/s). **D.** Traverse duration. **E.** Turn error rates of traverses. **F.** Tile errors of traverses. **G.** Corridor errors of traverses.

bioRxiv preprint doi: https://doi.org/10.64898/2026.08.30.747945; this version posted September 29, 2026. The copyright holder for this preprint (which was not certified by peer review) is the author/funder, who has granted bioRxiv a license to display the preprint in perpetuity. It is made available under a CC-BY 4.0 International license.

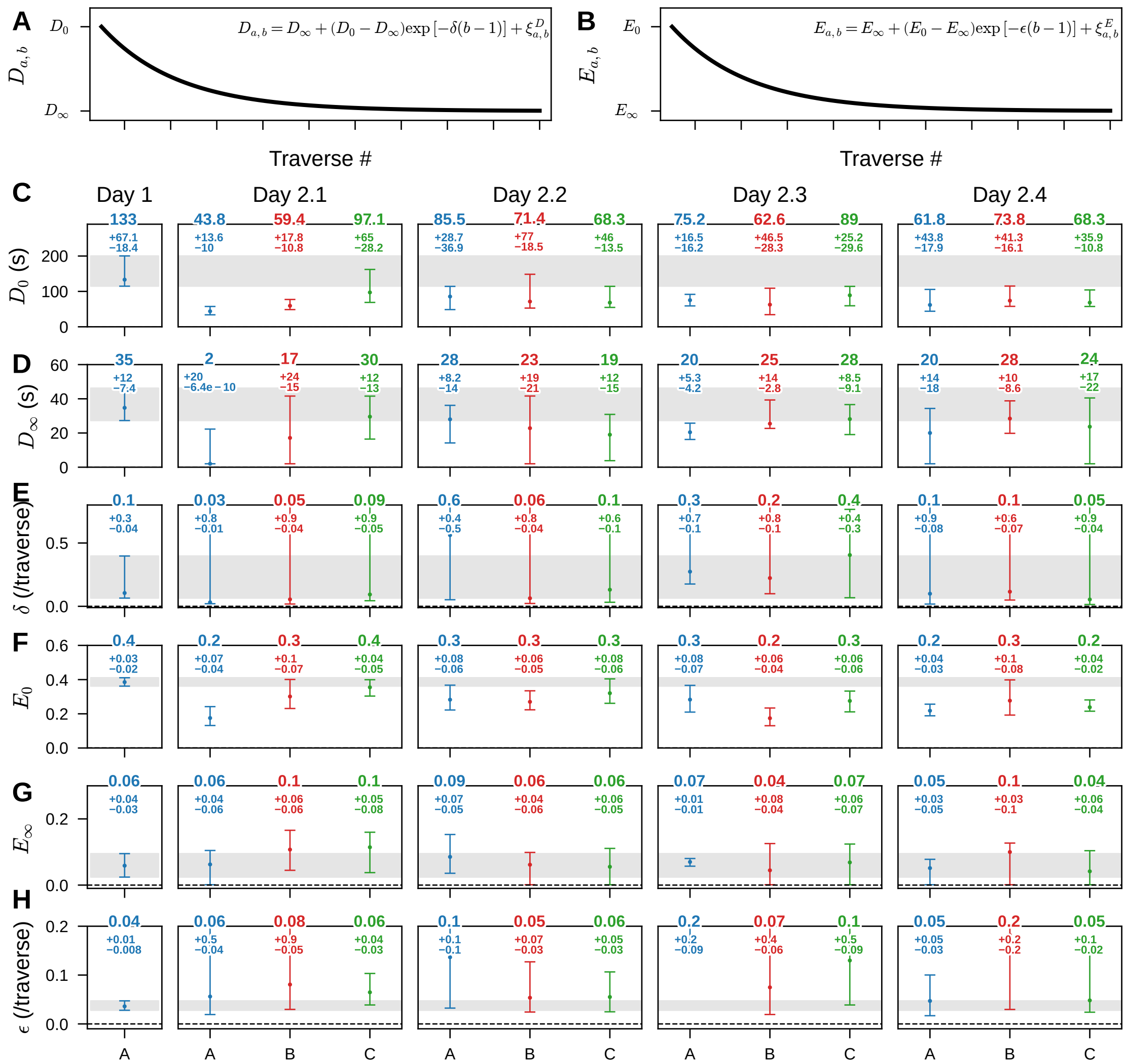

Supplementary Figure S3: **Confidence intervals of curve-fit parameters in the two-day experiments. A-B.** Equations and schematics of the fitted learning curves (See Section 5.6.4). **C-H.** Mask parameters are differentiated by colors: Blue for Mask A, red for Mask B, and green for Mask C. Numbers denote the point estimate, with 95% confidence intervals shown as asymmetric upper and lower deviations. The gray shades show the 95% confidence intervals of the parameters from Day 1. **C-E.** Parameter fits for traverse duration. **C.** D_0: duration of the first traverse in seconds. **D.** D_∞: asymptotic traverse duration in seconds. **E.** δ: learning rate. **F-H.** Parameter fits for traverse turn error rates. **F.** E_0: turn error rate of the first traverse. **G.** E_∞: asymptotic turn error rate. **H.** ϵ: learning rate.

bioRxiv preprint doi: https://doi.org/10.64898/2026.08.30.747945; this version posted September 29, 2026. The copyright holder for this preprint (which was not certified by peer review) is the author/funder, who has granted bioRxiv a license to display the preprint in perpetuity. It is made available under a CC-BY 4.0 International license.

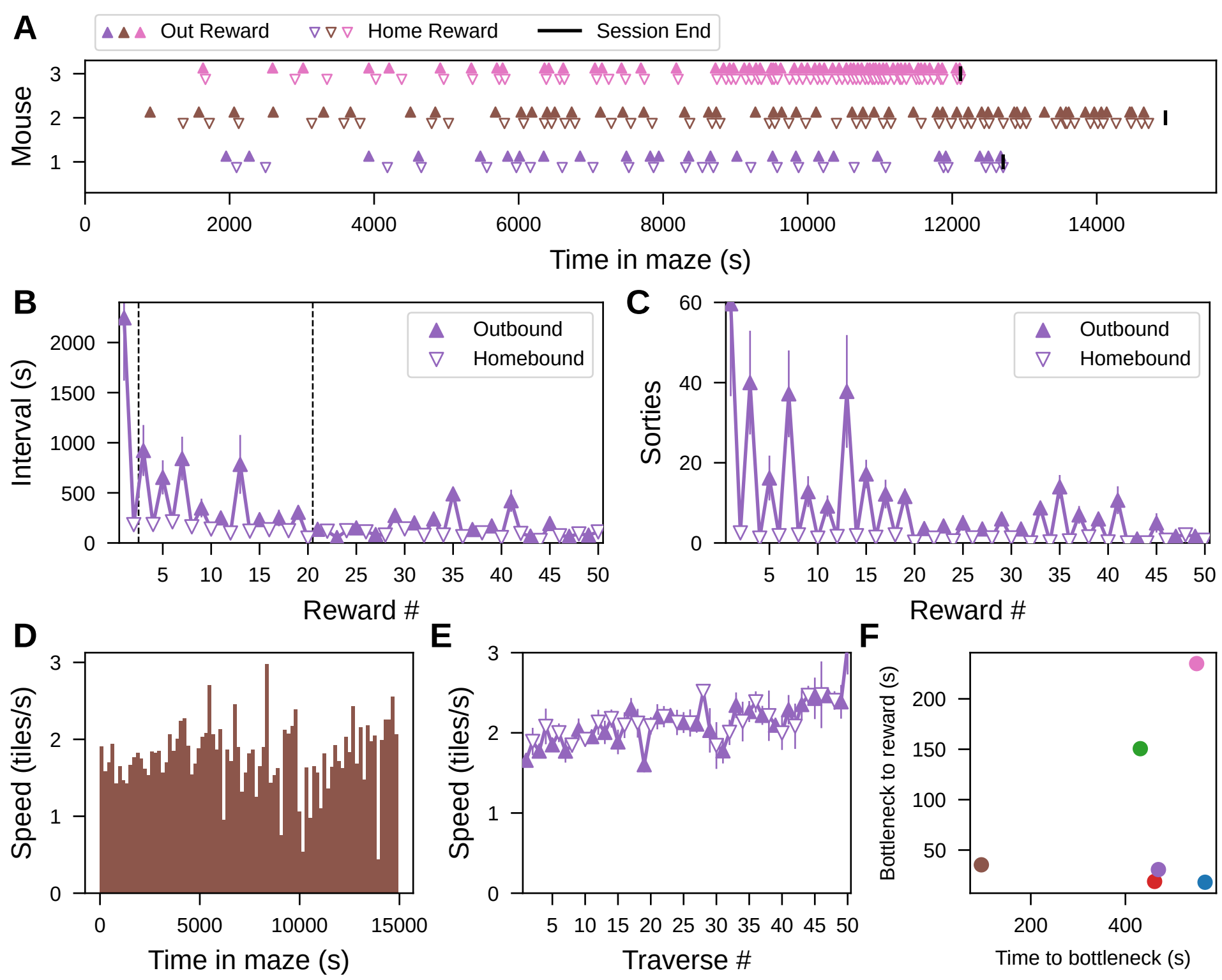

Supplementary Figure S4: **Additional metrics of learning Mask D as the first mask. A.** Rewards obtained over time in maze by three mice in the Mask D maze. Black vertical bars mark the end of sessions. **B.** Reward intervals (6 mice, mean ± SE across animals). **C.** Number of sorties between rewards (mean ± SE). **D.** Speed of Wildtype 1 in Panel A during the entire session. **E.** Traverse speeds of all mice. **F.** Time (in maze) to reach the bottleneck for the first time vs. time (in maze) between the last visit of the bottleneck and the first reward. Points are individual animals.

bioRxiv preprint doi: https://doi.org/10.64898/2026.08.30.747945; this version posted September 29, 2026. The copyright holder for this preprint (which was not certified by peer review) is the author/funder, who has granted bioRxiv a license to display the preprint in perpetuity. It is made available under a CC-BY 4.0 International license.

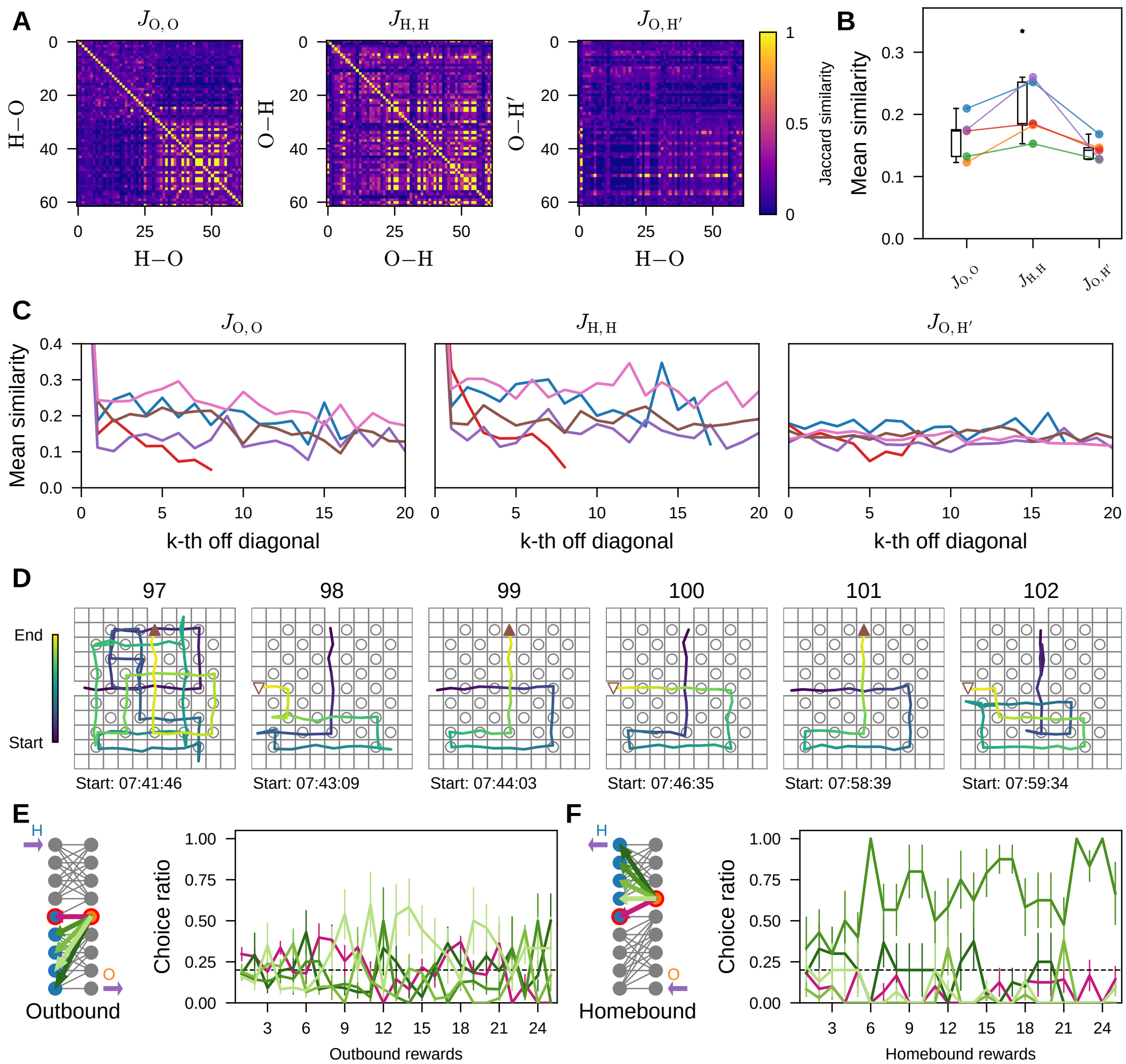

Supplementary Figure S5: **Varied routes in the Mask D maze. A.** The Jaccard similarity (Section 5.6.7) between the transition vectors in bicliques in outbound traverses ($J_{\mathrm{O,O}}$), homebound traverses ($J_{\mathrm{H,H}}$), and traverse pairs ($J_{\mathrm{O,H'}}$) by the mouse in Fig. 3B. **B.** Mean similarity values off-diagonal for all three groups of vectors (5 mice). Each colored point is a unique animal (Friedman test, $\chi^2(2) = 8.40$, $p = 0.015$, paired Wilcoxon signed-rank test not significant). **C.** Mean similarities over the distance off diagonal (each colored line is a unique animal that corresponds to B). **D.** Temporally adjacent traverses by the mouse in Fig. 3B. **E-F.** At the symmetric point of the bottleneck, preference for reversing back to the bottleneck (pink) compared to chance (horizontal dashed line, 0.2) and the other corridors (green).

bioRxiv preprint doi: https://doi.org/10.64898/2026.08.30.747945; this version posted September 29, 2026. The copyright holder for this preprint (which was not certified by peer review) is the author/funder, who has granted bioRxiv a license to display the preprint in perpetuity. It is made available under a CC-BY 4.0 International license.

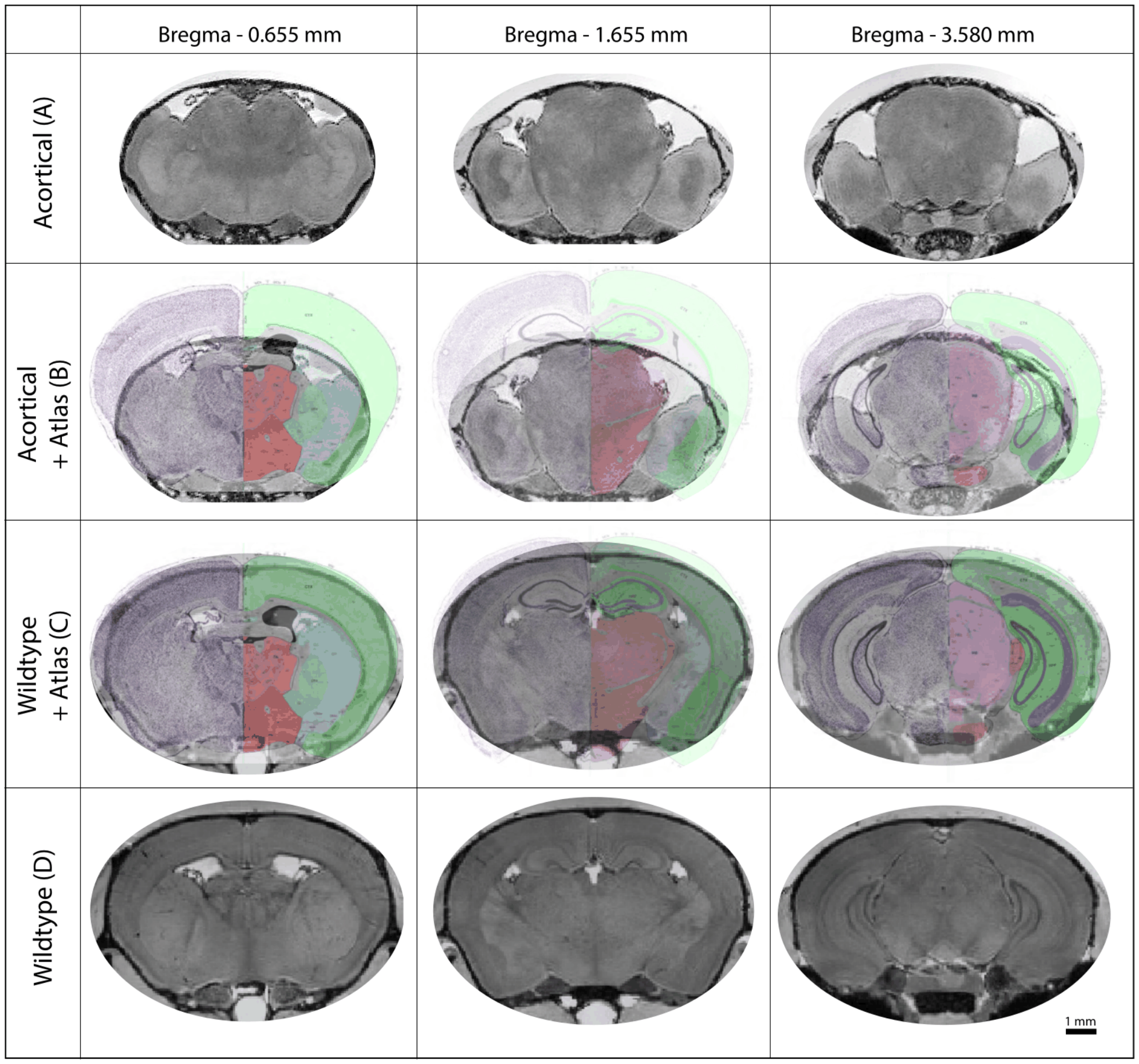

Supplementary Figure S6: **Mutant mouse with no neocortex and no hippocampus.** Coronal MRI sections at three anterior-posterior locations (columns): Bregma −0.655 mm, Bregma −1.655 mm, and Bregma −3.580 mm. The corresponding coronal sections are overlaid with atlas rows from the Allen Institute Mouse Brain Reference Atlas [73, 74]. Scale bar, 1 mm. **A.** Sections from an acortical mouse. **B.** The same sections as A with the atlas overlay. **C.** Sections from a wildtype mouse with the atlas overlay. **D.** The same wildtype sections without overlay.

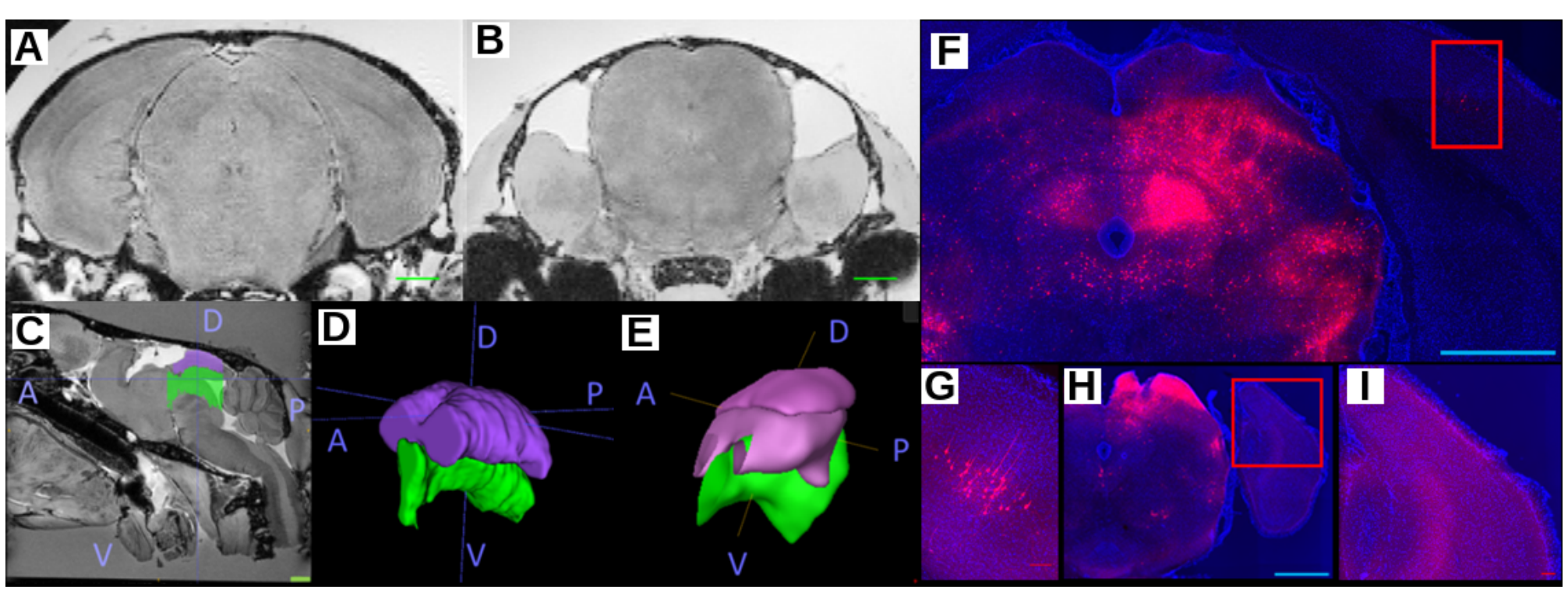

Supplementary Figure S7: **Midbrain structures of the mutant mouse. Superior colliculus in the mutant mouse does not receive cortical input. A-B.** High resolution T2*-weighted 3D gradient echo images of wildtype (A) and mutant (B) mice. **C.** Sagittal section of mutant mice showing superior colliculus (SC) in purple and periaqueductal gray (PAG) in green. Blue axes show anterior (A), posterior (P), dorsal (D) and ventral (V) directions. **D-E.** 3D reconstruction of SC and PAG in mutant (D) and wildtype mice (E); scale bar, 1 mm. **F-I.** Retrograde labeling of projection neurons in superficial SC via long term HSV-hEF1α-mCherry. SC in wildtype mice (F) receives projections from primary visual cortex (inset and G). Acortical mice (H) lack any cortical input (inset and I) to SC; scale bar, 1mm.

bioRxiv preprint doi: https://doi.org/10.64898/2026.08.30.747945; this version posted September 29, 2026. The copyright holder for this preprint (which was not certified by peer review) is the author/funder, who has granted bioRxiv a license to display the preprint in perpetuity. It is made available under a CC-BY 4.0 International license.

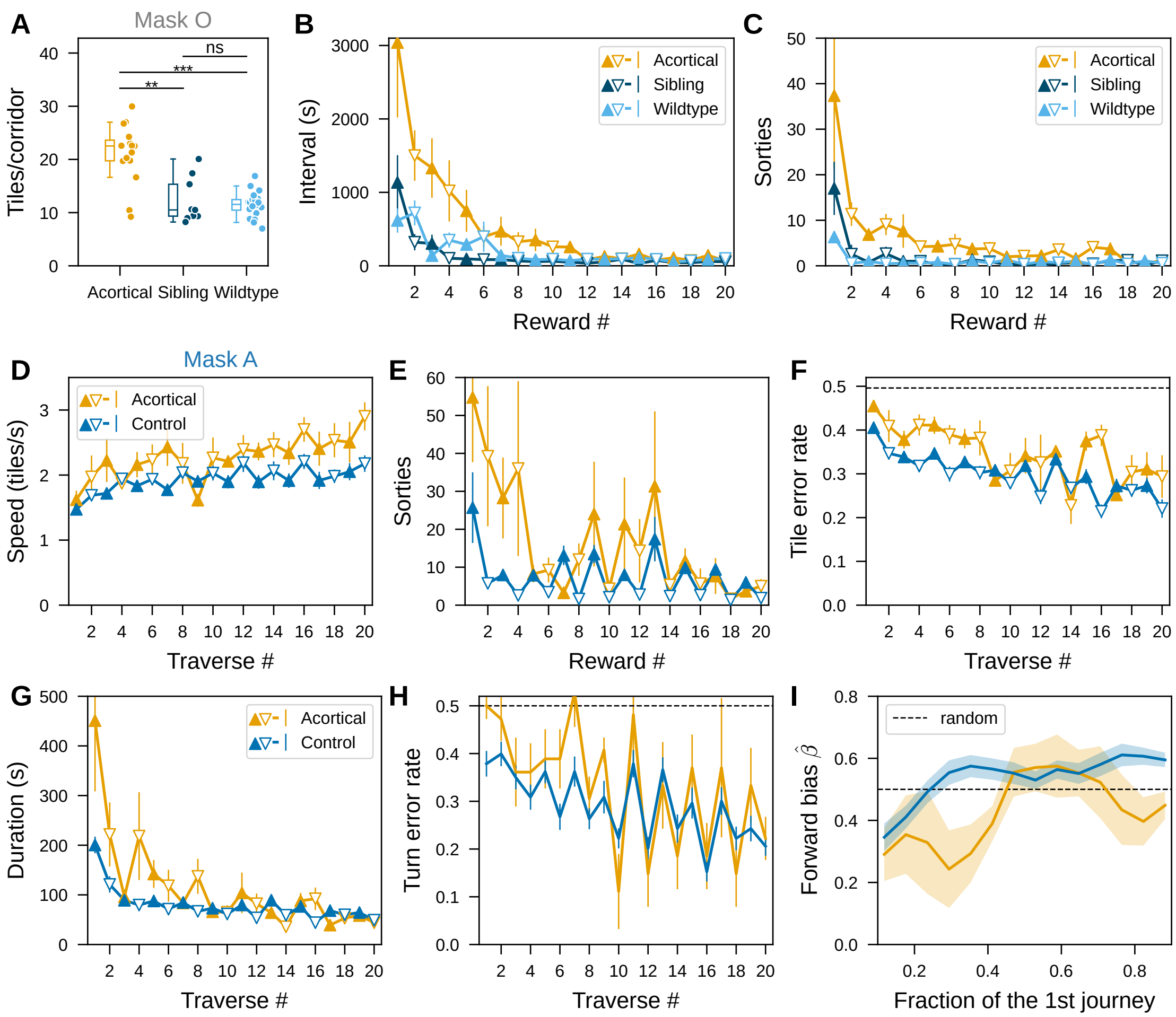

Supplementary Figure S8: **Slower initial learning of acortical mice.** Metrics comparing acortical (orange), sibling (dark blue) and wildtype (light blue) mice. Lines and error bars show mean ± SE across animals. **A-C.** In the Mask O Maze. **A.** Tiles per corridor in the first two rewards compared across acortical (15 mice, orange), sibling (9 mice, dark blue) and wildtype (25 mice, light blue). Asterisks mark significant two-sided pairwise Mann-Whitney U tests (acortical vs. sibling, $U = 119$, $p = 0.002$; acortical vs. wildtype, $U = 335$, $p = 4.0 \times 10^{-5}$; sibling vs. wildtype, $U = 105$, $p = 0.78$, n.s.) after a significant Kruskal-Wallis test ($H = 18.7$, $p = 8.8 \times 10^{-5}$). **B.** Reward intervals (session time) of acortical, sibling and wildtype mice. The light blue curve is identical to Fig. S1D. **C.** Number of sorties. **D-I.** In the Mask A maze. Control mice (blue) are pooled sibling and wildtype mice. **D.** Speed of traverses. **E.** Number of sorties. **F.** Tile error rate of traverses. Dashed line, chance error rate. **G.** Traverse duration. **H.** Turn error rate of traverses. Dashed line, chance error rate. **I.** Forward bias ($\hat{\beta}$, Section 5.7.1) along the first journey (mean ± SE). The dashed line marks the memoryless walker ($\beta = 0.5$). Lines smoothed with a moving average over 0.2 of the journey.

bioRxiv preprint doi: https://doi.org/10.64898/2026.08.30.747945; this version posted September 29, 2026. The copyright holder for this preprint (which was not certified by peer review) is the author/funder, who has granted bioRxiv a license to display the preprint in perpetuity. It is made available under a CC-BY 4.0 International license.

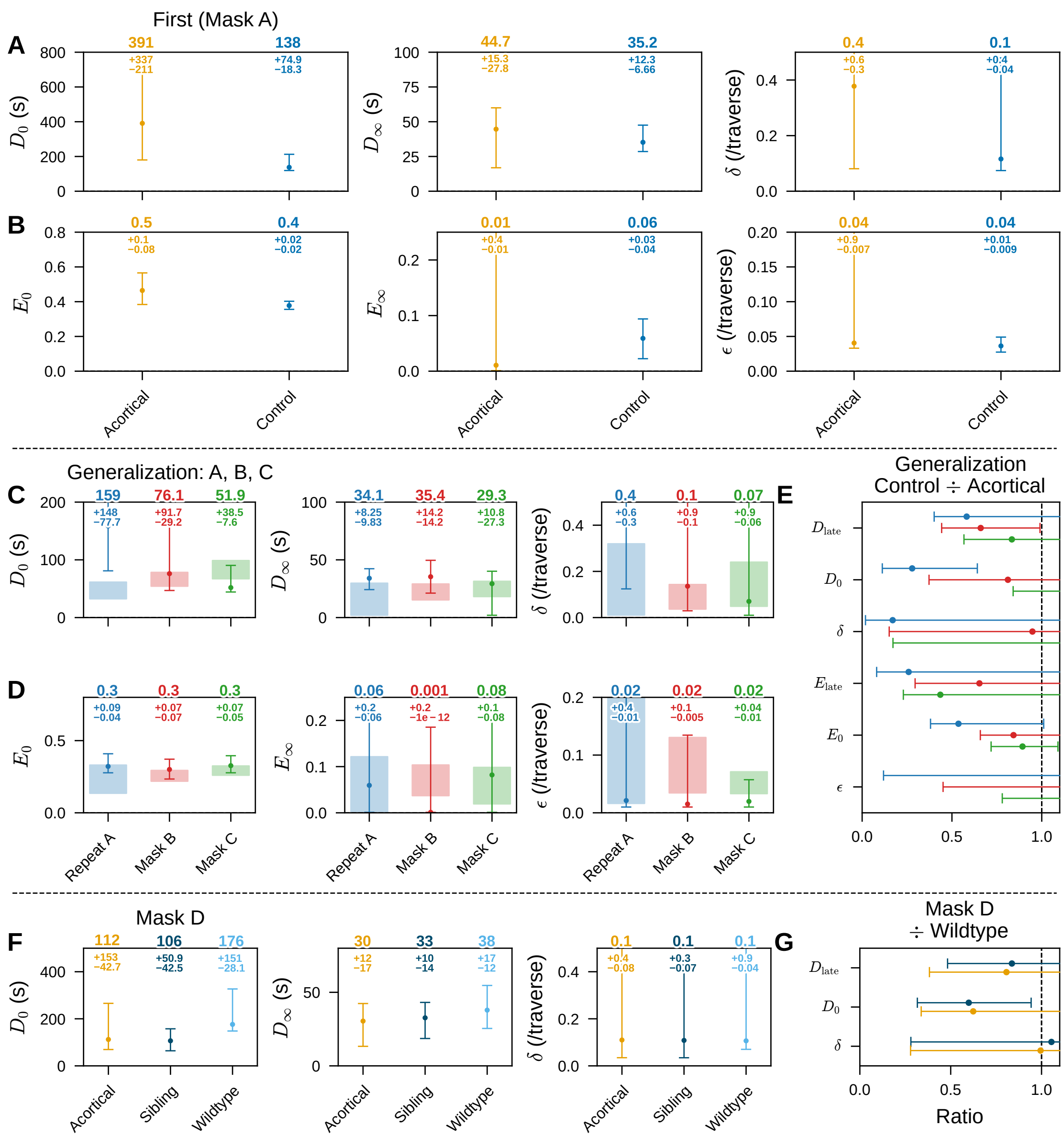

Supplementary Figure S9: **Curve fit results between acortical and control mice.** Numbers denote the point estimate, with 95% confidence intervals shown as asymmetric upper and lower deviations. **A-B.** Learning Mask A as the first mask across acortical (4 mice, orange) and control (27 mice, blue). **A.** Parameter fits for traverse duration. D_0: duration of the first traverse in seconds. D_∞ duration of the optimal traverse in seconds. δ learning rate. **B.** Parameter fits for traverse turn error rates. E_0: turn error rate of the first traverse. E_∞ turn error rate of the optimal traverse. ϵ learning rate. **C-D.** Exponential fit parameters for traverse duration (C) and turn error rate (D) on new masks and repeated Mask A. The shaded regions plot the same parameter fits for the control group: pooled for Mask B (28 mice) and Mask C (31 mice), and sibling mice alone for the repeated Mask A (7 mice). **E.** Generalization parameter ratios for both traverse duration and turn error rate across the repeated Mask A, Mask B and Mask C, computed as control/acortical. Each marker is the bootstrap median ratio and its 95% percentile confidence interval (Section 5.6.5); a ratio of 1 (dashed line) indicates no difference between cohorts. **F.** Exponential fit parameters for traverse duration in the Mask D maze across acortical (5 mice, orange), sibling (8 mice, dark blue) and wildtype (6 mice, light blue). **G.** As in E, Mask D duration-parameter ratios relative to wildtype: sibling/wildtype (dark blue) and acortical/wildtype (orange).

bioRxiv preprint doi: https://doi.org/10.64898/2026.08.30.747945; this version posted September 29, 2026. The copyright holder for this preprint (which was not certified by peer review) is the author/funder, who has granted bioRxiv a license to display the preprint in perpetuity. It is made available under a CC-BY 4.0 International license.

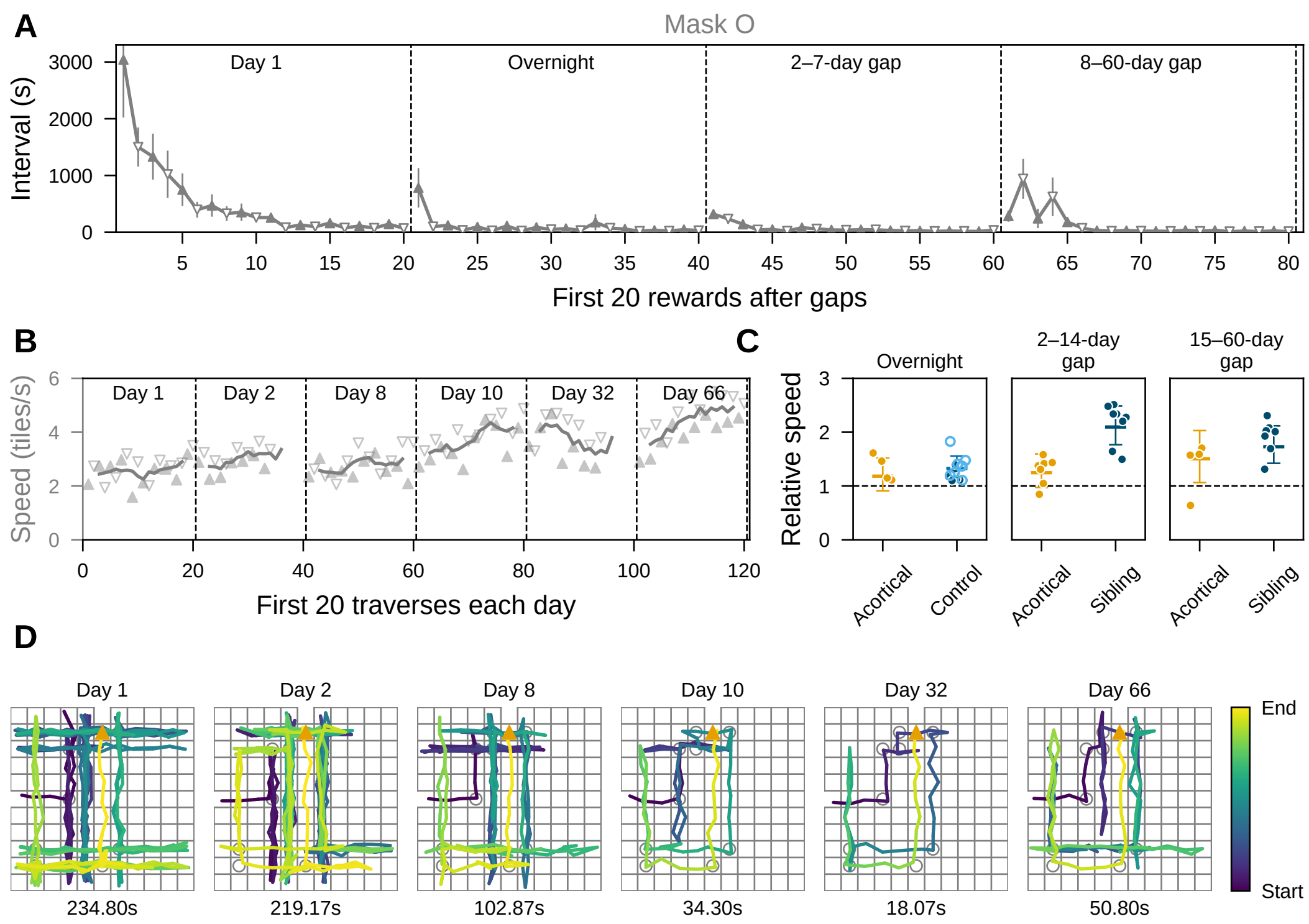

Supplementary Figure S10: **Long-term memory of masks.** **A.** Memory of Mask O in acortical mice (14 mice). Reward intervals (session time) of acortical mice in their first session overnight and after 2-7 days or 8-60 days of gap. The first panel is identical to the orange curve in Fig. S8B. **B.** Traverse speed (tiles/s) of the mouse in Fig. 5A. Solid lines are moving average over five traverses. **C.** Relative mean traverse speed in acortical (6 mice, orange), sibling (8 mice, dark blue), and wildtype (8 mice, light blue). The error bar shows 95% confidence intervals of the population-level mean, calculated from the pooled traverses across all sessions within each gap range (see text). Dots are from individual sessions. **D.** First traverse on each day in the Mask A maze by mouse in Fig. 5A.

bioRxiv preprint doi: https://doi.org/10.64898/2026.08.30.747945; this version posted September 29, 2026. The copyright holder for this preprint (which was not certified by peer review) is the author/funder, who has granted bioRxiv a license to display the preprint in perpetuity. It is made available under a CC-BY 4.0 International license.

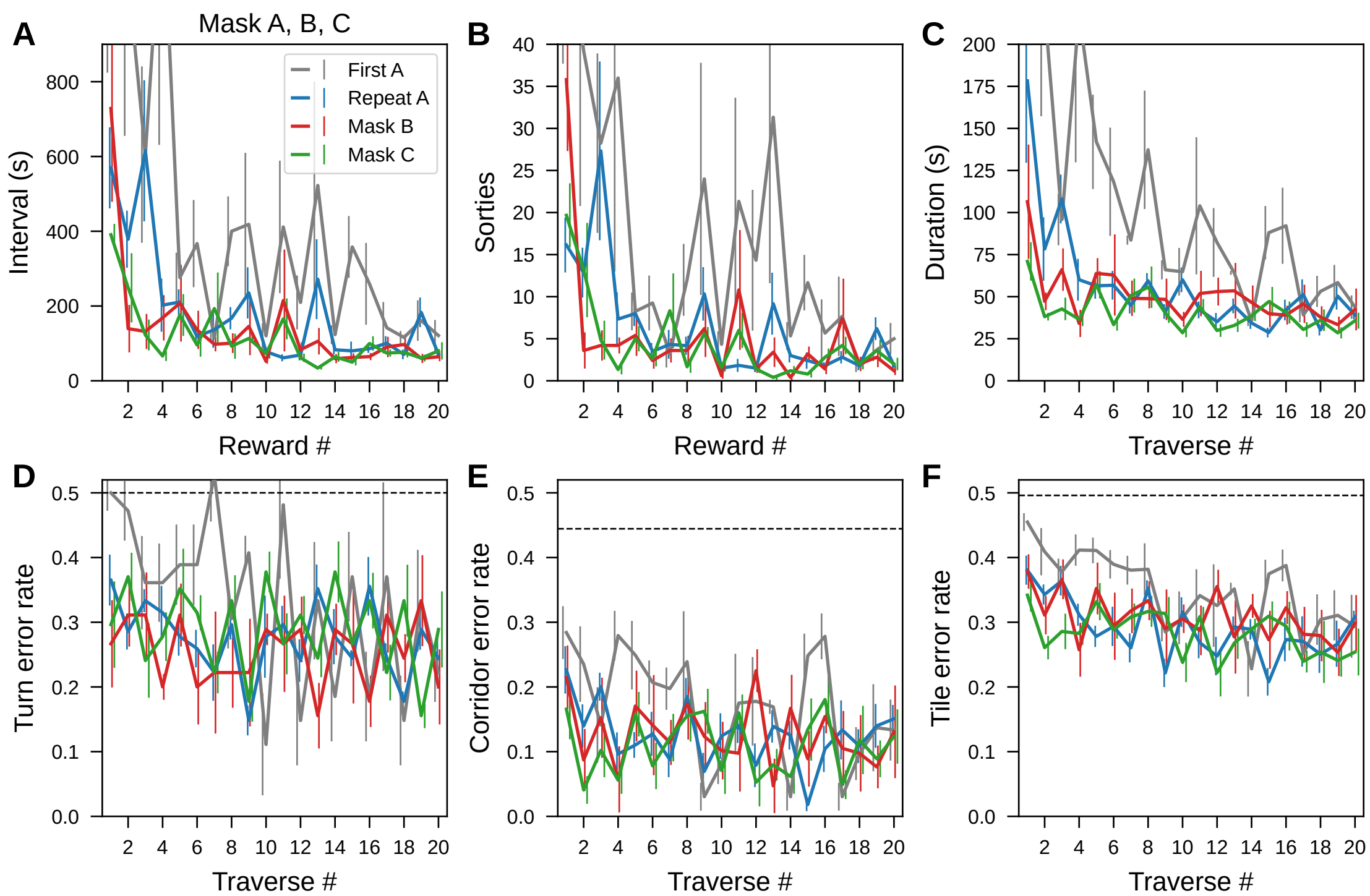

Supplementary Figure S11: **Acortical mice generalized to Masks B and C. A.** Reward intervals on new masks (Masks B and C) and repeated Mask A, compared to the first time in the Mask A maze (gray). **B.** Number of sorties between rewards. Gray curve is identical to the orange curve in Fig. S8E. **C.** Traverse duration. Gray curve is identical to the orange curve in Fig. S8G. **D.** Turn error rate of traverses. Gray curve is identical to the orange curve in Fig. S8H. Dashed line, chance error rate (0.5). **E.** Corridor error rate of traverses. Dashed line, chance error rate (0.44). **F.** Tile error rate of traverses. Dashed line, chance error rate (0.50).

bioRxiv preprint doi: https://doi.org/10.64898/2026.08.30.747945; this version posted September 29, 2026. The copyright holder for this preprint (which was not certified by peer review) is the author/funder, who has granted bioRxiv a license to display the preprint in perpetuity. It is made available under a CC-BY 4.0 International license.

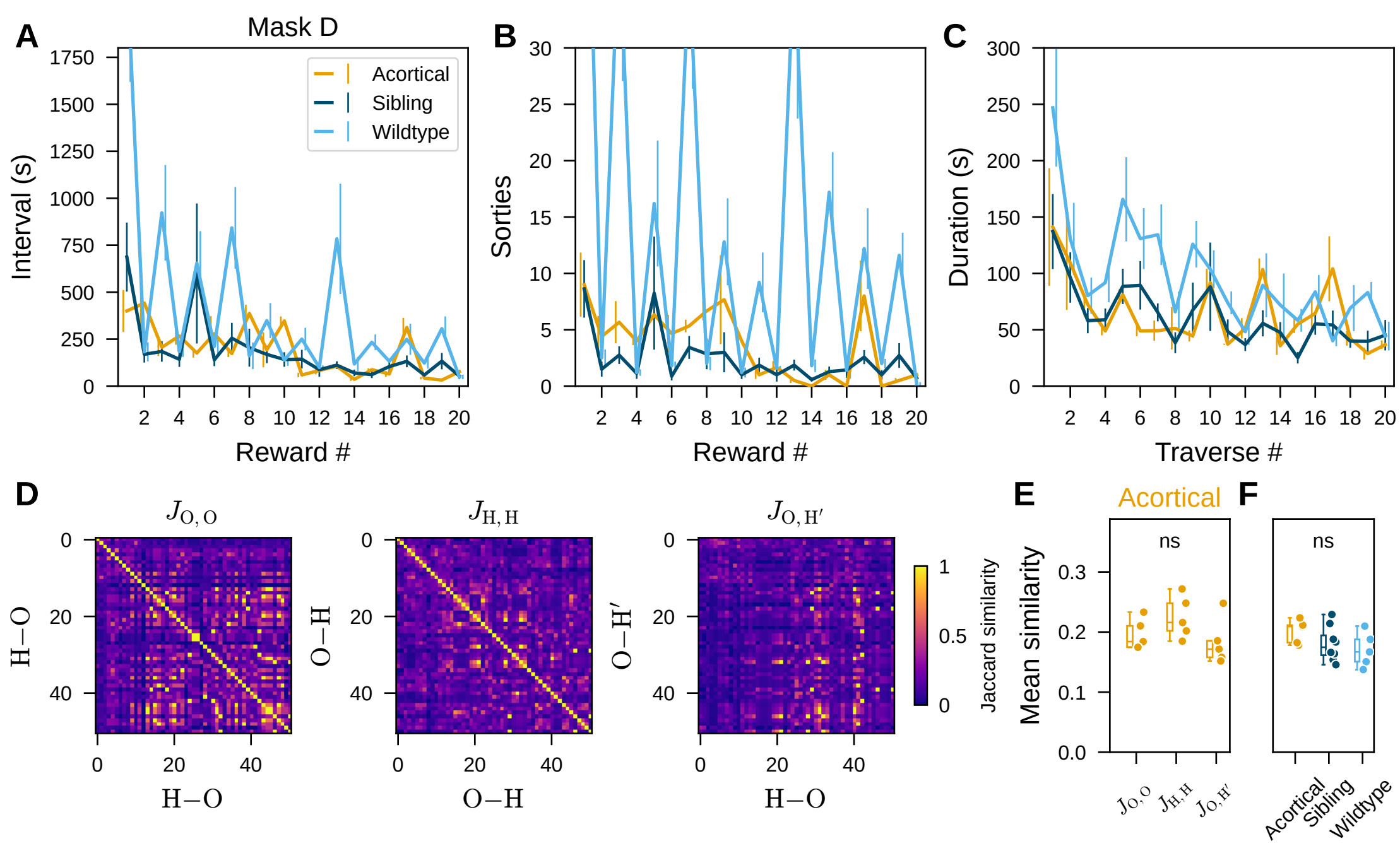

Supplementary Figure S12: **Acortical mice generalized to Mask D without repeating a fixed route. A-C.** Mask D learning in acortical (5 mice, orange), sibling (8 mice, dark blue) and wildtype (6 mice, light blue); lines and error bars show mean ± SE across animals. **A.** Reward intervals. The light blue curve is the first 20 rewards of Fig. S4B. **B.** Number of sorties. The light blue curve is the first 20 rewards of Fig. S4C. **C.** Traverse duration. The light blue curve is the first 20 traverses of Fig. 3C. **D.** The three similarity matrices of one example acortical mouse, plotted as in Fig. S5A. **E.** Adjusted-Jaccard route similarity within and between traverse directions, acortical mice (5 mice; Friedman test not significant, $\chi^2(2) = 5.20$, $p = 0.074$). **F.** Mean route similarity per animal, compared across genotypes, restricted to animals scored in all three similarity groups (acortical 5, sibling 8, wildtype 5; Kruskal-Wallis test not significant, $H = 2.44$, $p = 0.29$).

bioRxiv preprint doi: https://doi.org/10.64898/2026.08.30.747945; this version posted September 29, 2026. The copyright holder for this preprint (which was not certified by peer review) is the author/funder, who has granted bioRxiv a license to display the preprint in perpetuity. It is made available under a CC-BY 4.0 International license.

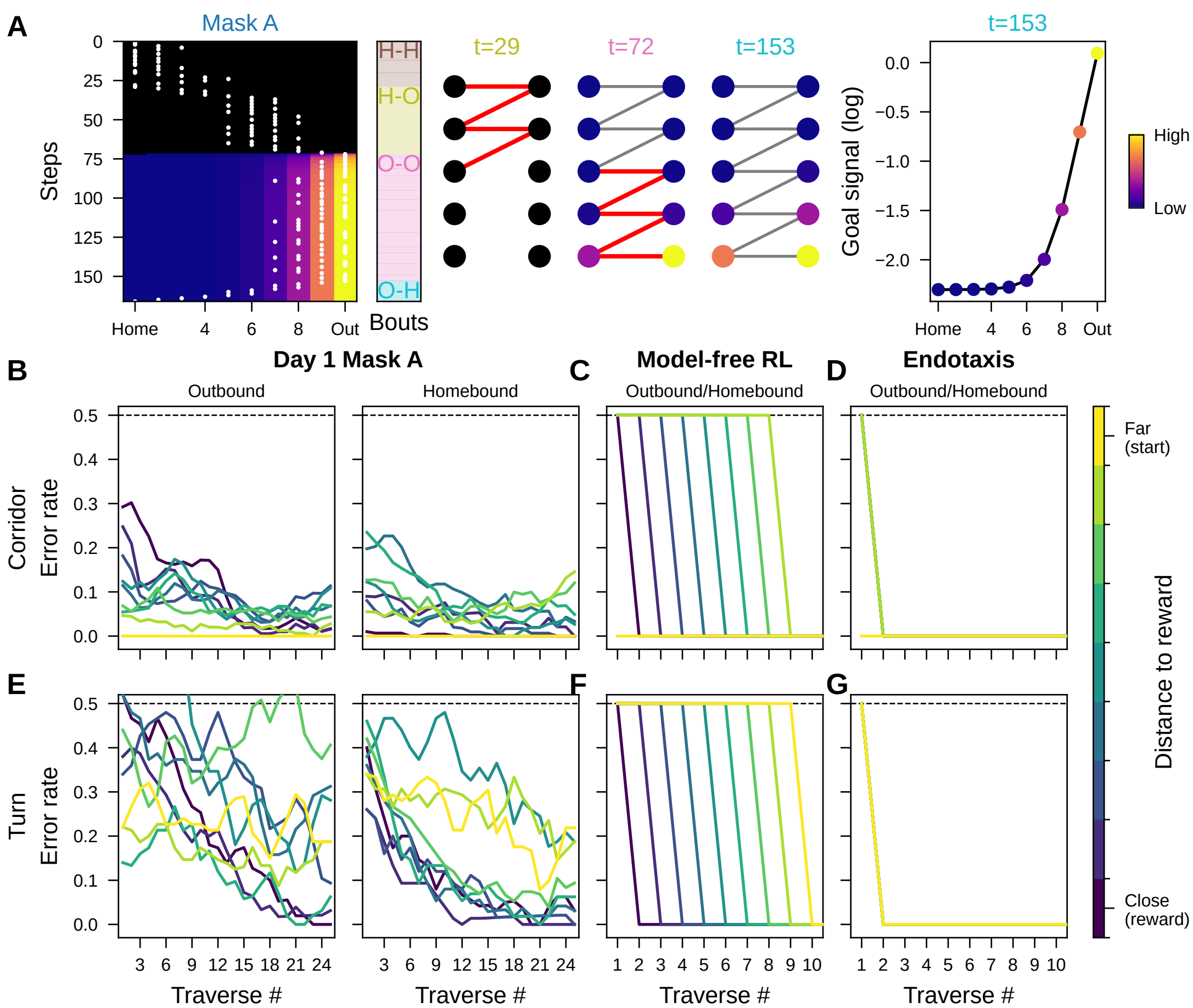

Supplementary Figure S13: **Mouse error propagation in the Mask A maze differs from model-free RL and Endotaxis predictions. A.** Endotaxis model in the Mask A corridor graph, trained on the first two journeys of the mouse in Fig. 1E, as in Fig. 6. Left: propagation of the goal signal through corridor visits. Middle: goal signals and learned adjacency on the Mask A corridor graph, with newly added edges in red. Right: goal signal at the last timepoint ($t = 153$). **B-G.** Error rate propagation. Throughout, each line is one path position, colored by distance to reward from close (dark) to far (light); the dashed line marks chance (0.5). **B.** Corridor error rate per corridor across traverses, as the population mean over all 25 animals in Fig. 1G (smoothed with a 3-traverse moving-average window). **C.** Analytic prediction of the model-free RL agent for the same corridor error rate. **D.** Analytic prediction of the Endotaxis model for the same corridor error rate. **E-G.** As in **B-D**, for turn error rate per hole.

bioRxiv preprint doi: https://doi.org/10.64898/2026.08.30.747945; this version posted September 29, 2026. The copyright holder for this preprint (which was not certified by peer review) is the author/funder, who has granted bioRxiv a license to display the preprint in perpetuity. It is made available under a CC-BY 4.0 International license.

6.2 Supplementary videos

Video V1. Manhattan Maze experiment setup. Overview of the recording arena and task structure, followed by trimmed raw footage of the maze being reconfigured from Mask O to Mask A. The outbound and homebound traverses in the Mask O maze illustrate the mouse alternating between the two water ports (Fig. 1A and Fig. S1A-D). Shown at 2× speed.

Video V2. First bouts of a wildtype mouse in the Mask A maze. Continuous segment spanning the first 29 bouts of a wildtype mouse's initial exposure to Mask A, including both traverses and sorties, with a scrolling inset of tile distance to the rewarded port (Fig. 1E). Shown at 8× speed.

Video V3. Overnight memory in the Mask A maze. Outbound and homebound traverses from Day 1 and Day 2.1 of the same mouse, each rendered individually with a growing inset of tile distance to reward colored to match the figure, illustrating overnight memory retention (Fig. 2C). Shown at 4× speed.

Video V4. Late bouts in the Mask D maze. Continuous segment of six consecutive traverses late in learning of the Mask D maze, including the interleaved sorties, with a bout trajectory inset (Fig. S5D). Shown at 8× speed.

Video V5. Acortical mouse learning Mask A. Continuous segment of an acortical mouse learning Mask A, including both traverses and sorties, with a scrolling inset of tile distance to the rewarded port (Fig. 4B). Shown at 8× speed.

Video V6. Acortical and wildtype mouse late traverses in the Mask A maze. The 19th and 20th Mask A traverses of an acortical mouse compared to the reference traverses of a wildtype mouse, each rendered individually with a growing inset of tile distance to reward colored to match the figure (Fig. 4D). The wildtype traverses are identical to the two in Video V3. Shown at 4× speed.

6.3 Maze construction and maintenance

The physical mazes in this study used a two-layered 11×11 structure (Fig. 1A and Fig. S1A). Each maze apparatus was made of clear acrylic (ePlastics, San Diego). The two layers were two identical, ∼18-inch square open-top trays of eleven 1.5-inch wide and 1.5-inch high corridors (wall thickness not included), with one access hole (1.5-inch diameter) on the side wall of the central corridor. We slotted the bottom plate (0.25 inches thick) of a tray every 1.5 inches, and then inserted ten walls (0.118 inches thick) into the slots to separate the tray into eleven corridors. The slots were laser cut to ensure that all eleven corridors were equally wide. The total width of the square tray was ∼18 inches, depending on the precise thickness of the materials. To form a maze, the two identical trays were stacked with bottom plates facing out, creating a closed space within the maze that could only be entered through the access holes. The top tray was rotated 90 degrees from the bottom tray, making the corridors in the two layers perpendicular. From the top view, the walls created an 11×11 grid pattern.

Between the two trays was a square mask with a series of holes. All masks were 0.125 inches thick, and the holes were of the same diameter (1.25 inches). Each hole was laser cut and centered at its corresponding grid coordinate. The masks were also made of clear acrylic, allowing imaging through the whole assembly via a top camera.

The mazes were sanitized regularly to remove odor and bacteria. The masks were wiped with disinfectants and deodorizers (Peroxigard, 1:16) before usage. After each day's experiment, the maze trays were also cleaned thoroughly with deodorizers. All acrylic compartments contacted by the animals were submerged in room-temperature cleaning solution once per week. All items passed the annual NovaLum swab tests for sanitation verification.

6.4 Acortical mice learned intermediate masks and generalized

Because many acortical mice struggled with reward discovery in the more difficult masks, we tested whether they could learn an intermediate maze configuration. After learning Mask O, a subset of acortical mice was introduced to Mask E, a path graph with four corridors and three turns (Fig. S14A). These mice were initially unwilling to traverse through an additional hole and often became trapped in the vertical corridors. Therefore, they were guided through the holes until they connected movements across all corridors (Section 5.5.3). With this assisted training, 10 of 11 acortical mice learned Mask E (Table 2).

bioRxiv preprint doi: https://doi.org/10.64898/2026.08.30.747945; this version posted September 29, 2026. The copyright holder for this preprint (which was not certified by peer review) is the author/funder, who has granted bioRxiv a license to display the preprint in perpetuity. It is made available under a CC-BY 4.0 International license.

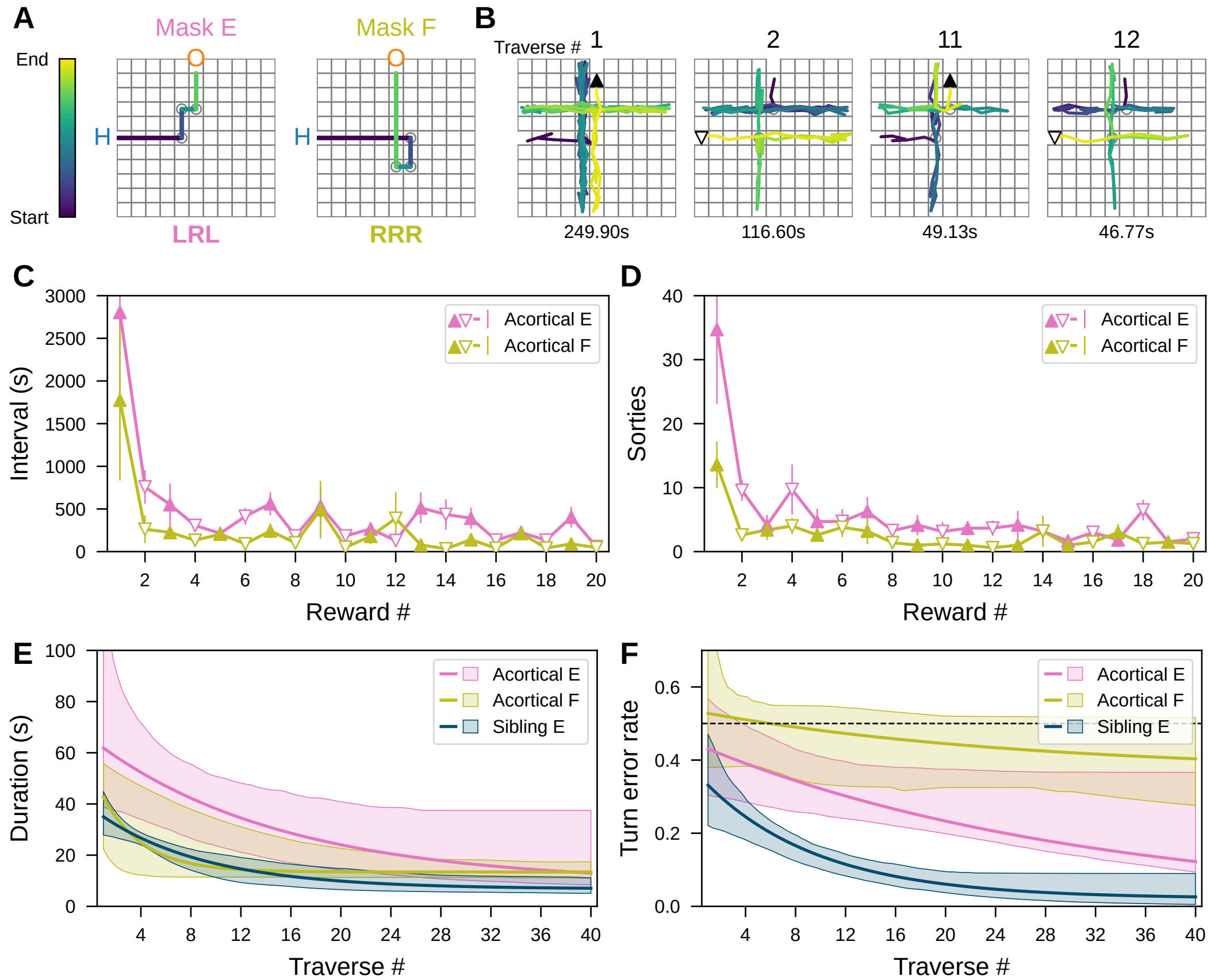

Supplementary Figure S14: **Acortical mice generalized from Mask E to Mask F. A.** Top view of Mask E and Mask F. Of each mask, the shortest path is a sequence of three turns. **B.** Example traverses from an acortical mouse in the Mask E maze (text shows traverse duration). **C.** Reward intervals (session time) on Mask E (7 mice, pink) vs. Mask F (5 mice, olive, mean ± SE). **D.** Number of sorties between rewards (mean ± SE). **E.** Exponential fits of traverse duration for Mask E and Mask F, compared with sibling mice (6 mice, dark blue). **F.** Exponential fits of turn error rates of traverses. Dashed line, chance error rate.

Despite the simplicity of Mask E relative to Mask A, acortical mice still showed impaired initial performance. Their first traverses were prolonged by repetitive scanning (Fig. S14B,D). However, performance improved rapidly over the next 10 rewards: reward intervals shortened to ∼0.15 of the initial value (Fig. S14C). Consistent with the results of Mask A, acortical mice began with longer traverses than sibling mice but eventually reached similar asymptotic traverse durations (Fig. S14E), although their turn error rates remained higher than those of sibling mice (Fig. S14F).

We then tested whether acortical mice could transfer this experience to a related maze. Seven acortical mice were introduced to Mask F, a center-symmetric variant of Mask E (Fig. S14A). In contrast to their difficulty with Mask E, five mice learned Mask F without assistance. They obtained the first reward in ∼0.6 of the time required in the Mask E maze (Fig. S14C) and started with shorter traverse durations (Fig. S14E). Together, these results show that acortical mice can learn multiple maze configurations and that prior experience can facilitate learning of a related path graph.

6.5 Massive landmark changes did not reset turn learning

To test whether landmarks in the Manhattan Maze contribute to navigation, we performed a tray-swapping manipulation (Fig. S15A). If a mouse marks the maze with scent or urine, these deposits are likely to end up on the bottom tray or on the mask, so swapping the two trays and replacing the mask should severely disrupt such markers. In addition, any other markings, such as inadvertent construction glitches that identify a specific corridor, were perturbed as well.

Eight animals were first trained in the Mask O maze and then introduced to Mask A as in the main training protocol (Section 5.5.2). After each mouse obtained ∼20 rewards in the Mask A maze, we temporarily trapped it in the home

bioRxiv preprint doi: https://doi.org/10.64898/2026.08.30.747945; this version posted September 29, 2026. The copyright holder for this preprint (which was not certified by peer review) is the author/funder, who has granted bioRxiv a license to display the preprint in perpetuity. It is made available under a CC-BY 4.0 International license.

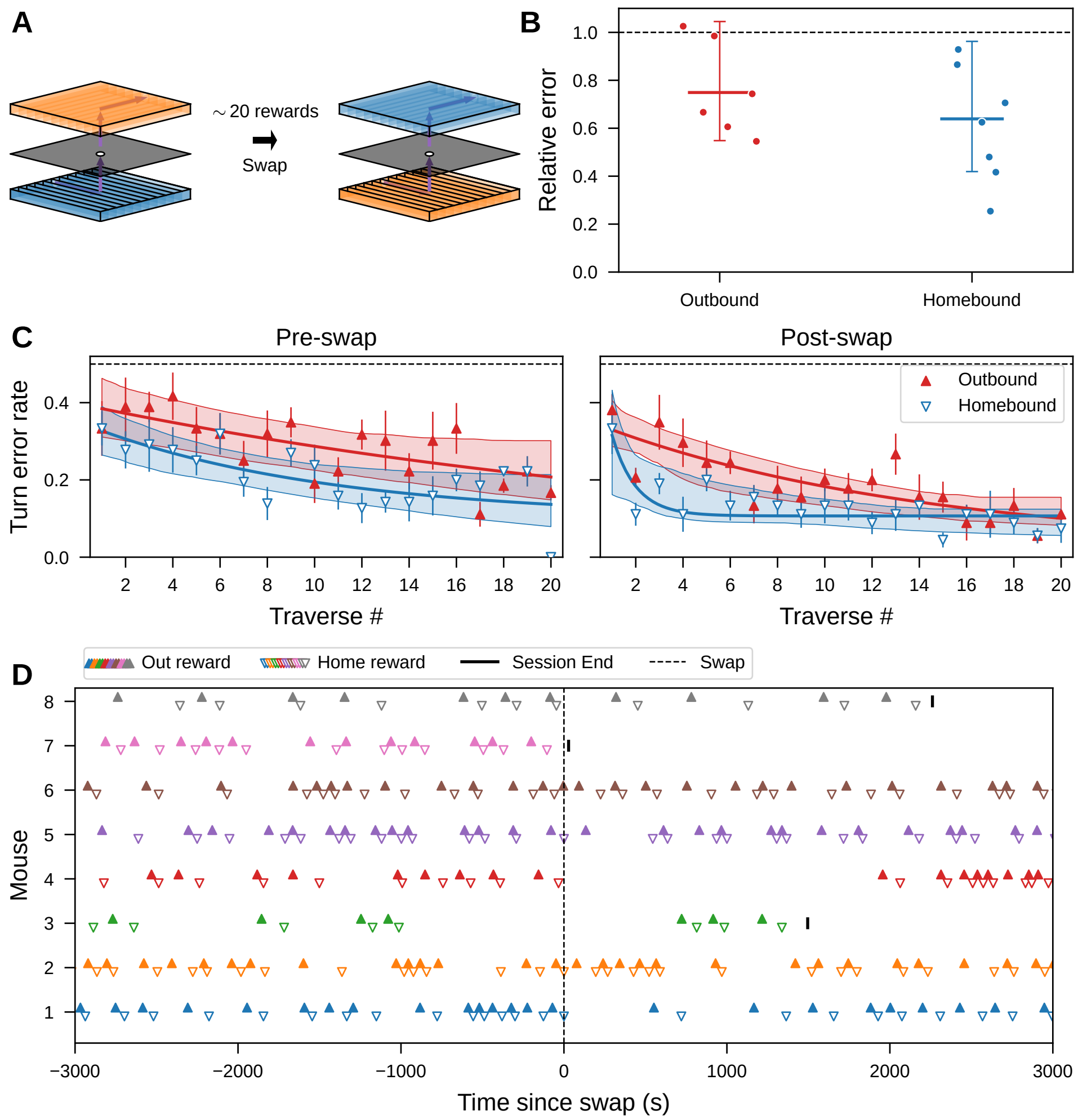

Supplementary Figure S15: **Mice quickly resumed learning after swapping the trays. A.** The swapping procedure: after each mouse obtained ∼20 rewards in the Mask A maze, the top and bottom trays were exchanged and the mask was replaced with a clean, identical copy. **B.** Relative mean turn error rates of the first 10 traverses post-swap compared with the first 10 traverses in the pre-swap session, shown separately for outbound (red) and homebound (blue) directions (8 mice). The dashed line at 1 marks complete reset (values below 1 indicate savings). Error bars show 95% confidence intervals of the population-level mean (see text). Dots are individual animals. **C.** Outbound (red, filled triangles) and homebound (blue, open triangles) turn error rates before (left) and after (right) the swap. Points and error bars show population mean $\pm$ SE; solid lines are exponential fits of the population and shaded bands are their 95% bootstrap confidence intervals. Dashed line, chance error rate. **D.** Rasters of rewards of all mice in the experiment, aligned by the time (in maze) since the swap (dashed line at 0). Outbound (up triangles) and homebound (down triangles) rewards are shown per mouse; the pre-swap session runs in negative time and the post-swap session in positive time, and the solid line marks each session's end.

bioRxiv preprint doi: https://doi.org/10.64898/2026.08.30.747945; this version posted September 29, 2026. The copyright holder for this preprint (which was not certified by peer review) is the author/funder, who has granted bioRxiv a license to display the preprint in perpetuity. It is made available under a CC-BY 4.0 International license.

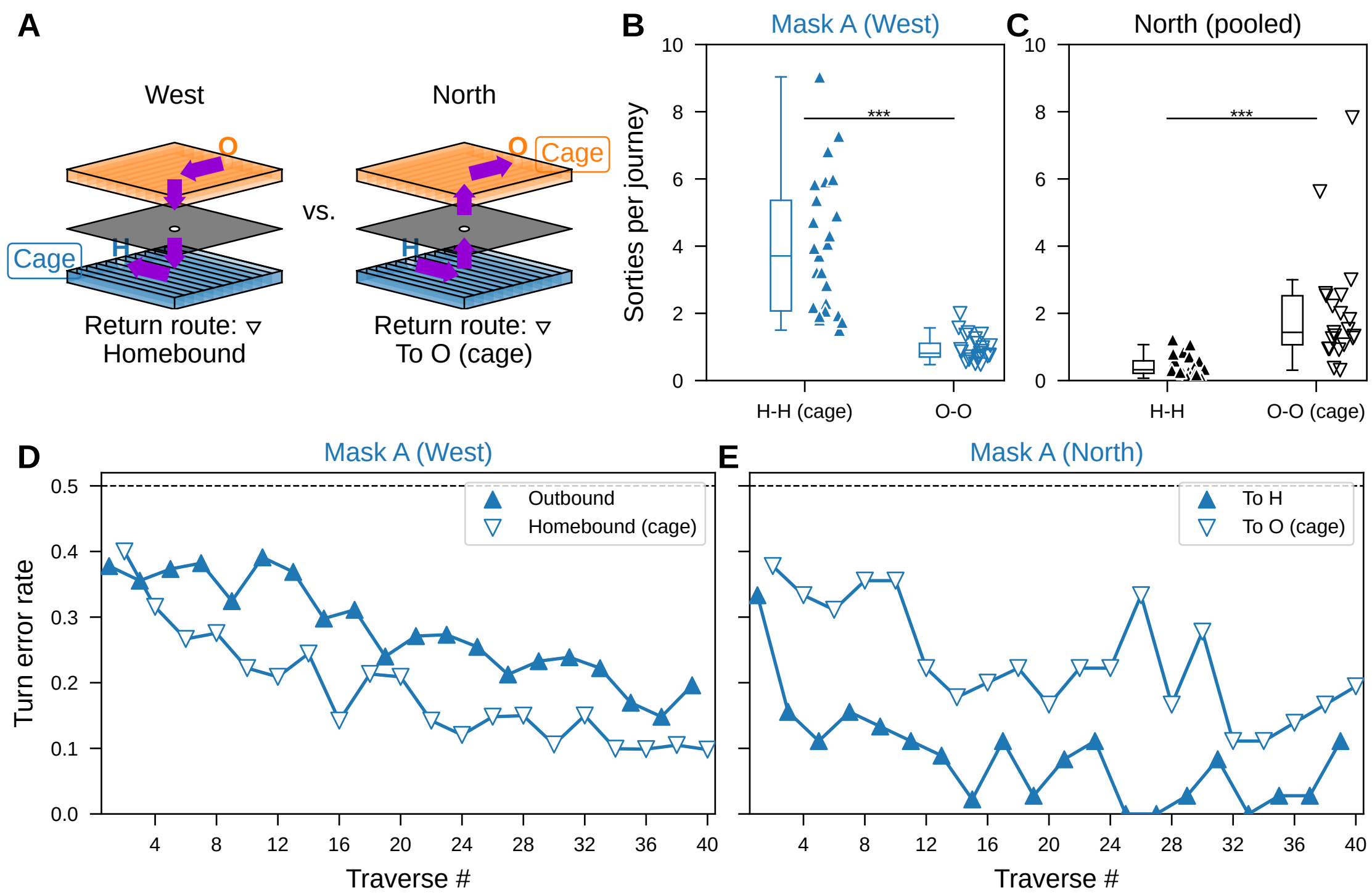

Supplementary Figure S16: **Maze navigation starting from O. A.** The two start configurations. Left ("West"): the original layout in the main text, with the home cage on the bottom tray at the H port. Right ("North"): the cage relocated to the top tray at the O port, so that the return route is directed to O. **B.** Mean number of sorties per journey split by port (H-H and O-O) in the Day-1 Mask A session ("West" layout, Fig. 1; 25 sessions from 25 mice, same data as Fig. 1G). Asterisks show two-sided within-subject paired Wilcoxon signed-rank test ($W = 0$, $p = 6 \times 10^{-8}$). **C.** As in **B**, in the North layout (21 sessions pooled across 6 mice; two-sided Wilcoxon signed-rank $W = 1$, $p = 1.9 \times 10^{-6}$). **D.** Turn error rate in the Day-1 Mask A session (25 mice, mean $\pm$ SE). Each direction is connected as a separate line. Traverses toward H and cage are labeled "Homebound" (filled up-triangles) and traverses toward O are labeled "Outbound" (open down-triangles). Dashed line, chance error rate (0.5). **E.** Turn error rate in the Mask A maze under the North condition (5 mice, mean $\pm$ SE). Traverses toward H are labeled "To H" (filled up-triangles) and traverses returning to the cage at O are labeled "To O (cage)" (open down-triangles).

cage, swapped the top and bottom trays of the two-layer maze, and inserted a clean copy of the Mask A plate. The home cage was then reconnected, allowing the mouse to resume exploration freely. The swap did perturb behavior transiently, but did not reset it: across the first ten post-swap traverses, turn errors were lower than in the first ten of the pre-swap session (Fig. S15B, methods from Section 5.6.5), most clearly for homebound turns, though they rose transiently over the first one or two post-swap traverses (Fig. S15C). One mouse did not enter the maze afterward, but 7 of 8 animals continued to obtain rewards (Fig. S15D). The swap thus did not produce a complete reset, and the animals did not have to relearn the turn sequences after the swap.

6.6 Outbound–homebound asymmetry was caused by route geometry

In the main experiments of Mask A, homebound traverses were less erroneous than the preceding outbound traverses (Fig. 1G). Two accounts could explain this asymmetry. First, the homebound direction might be learned faster because it is directed toward the home cage — a form of home-base behavior, in which rodents organize around the home base longer exploratory excursions away from home but shorter, more direct return trips [75]. Second, the two directions might simply be distinct physical routes whose geometry differs in difficulty, independent of cage location. In the Mask A maze, the outbound route requires climbing upward through 5 of 9 holes, whereas the homebound route descends. These accounts make opposite predictions when the cage is moved: a home-base advantage should follow the cage, so that the faster, more accurate traverses become those directed toward the new cage location, whereas a geometric advantage should remain bound to the same physical route regardless of where the cage sits.

bioRxiv preprint doi: https://doi.org/10.64898/2026.08.30.747945; this version posted September 29, 2026. The copyright holder for this preprint (which was not certified by peer review) is the author/funder, who has granted bioRxiv a license to display the preprint in perpetuity. It is made available under a CC-BY 4.0 International license.

To dissociate them, a new cohort was tested with the home cage moved from the H port to the O port while the mask orientation was held fixed. Mice therefore entered the maze via the top tray, from the "North" direction and, on each rewarded traverse, first traveled toward H — the physical route that had been the homebound route in the main experiments (Fig. S16A). If the asymmetry were motivational, performance should now favor the To-O traverses that return to the relocated cage; if geometric, it should still favor the H-directed route.

Home-base behavior itself was evident in the unrewarded sorties (Fig. S16B,C). In both layouts, mice launched more sorties from the cage-side port than from the opposite port, and this bias moved with the cage: from the H side under the West layout to the O side under North. On the other hand, the asymmetry tracked the physical route, not the cage. During initial training, mice hesitated to descend through the first hole but readily reversed and climbed once lowered to the bottom tray, after which the H-directed route was traversed fluently. In the Mask A maze, To-H traverses were less error-prone, consistent with the homebound traverses in the main text condition (Fig. S16D,E). Therefore, the outbound–homebound asymmetry in traverse performance in the main text is best explained by route geometry rather than by a home-base advantage.
